# Йододефицитные заболевания: текущее состояние проблемы в Брянской области

**DOI:** 10.14341/probl12793

**Published:** 2021-08-03

**Authors:** Е. А. Трошина, Н. П. Маколина, Е. С. Сенюшкина, Л. В. Никанкина, Н. М. Малышева, А. В. Фетисова

**Affiliations:** Национальный медицинский исследовательский центр эндокринологии; Национальный медицинский исследовательский центр эндокринологии; Национальный медицинский исследовательский центр эндокринологии; Национальный медицинский исследовательский центр эндокринологии; Национальный медицинский исследовательский центр эндокринологии; Департамент здравоохранения Брянской области

**Keywords:** йодный дефицит, зоб, йододефицитные заболевания, йодированная соль

## Abstract

ОБОСНОВАНИЕ. Брянская область относится к регионам Российской Федерации, в наибольшей степени пострадавшим в результате аварии на Чернобыльской атомной электростанции 26 апреля 1986 г. В условиях хронического некомпенсированного дефицита йода в питании в первые месяцы после аварии происходил активный захват радиоактивного йода тканью щитовидной железы (ЩЖ), что неизбежно реализовалось в росте заболеваний ЩЖ у населения в последующем. В статье представлены результаты проведенного в мае 2021 г. специалистами ФГБУ «НМИЦ эндокринологии» Минздрава России контрольно-эпидемиологического исследования, направленного на оценку современного состояния йодной обеспеченности населения Брянской области.ЦЕЛЬ. Оценка йодной обеспеченности населения Брянской области.МАТЕРИАЛЫ И МЕТОДЫ. Исследование проводилось в общеобразовательных школах трех районов Брянской области (гг. Брянск, Новозыбков и Клинцы). В исследование были включены 337 школьников допубертатного возраста (8–10 лет), всем детям выполнено: измерение роста и веса непосредственно перед осмотром врача, включавшим пальпацию ЩЖ; ультразвуковое исследование (УЗИ) ЩЖ с использованием портативного аппарата LOGIQe (China) с мультичастотным линейным датчиком 10–15 МГц; определение концентрации йода в разовых порциях мочи. Качественное исследование на наличие йодата калия в образцах пищевой поваренной соли (n=344), полученной из домохозяйств и школьных столовых, осуществлялось на месте экспресс-методом.РЕЗУЛЬТАТЫ. По результатам обследования 337 детей допубертатного возраста медианная концентрация йода в моче составляет 98,3 мкг/л (диапазон от 91,5 до 111,5 мкг/л, доля проб мочи со сниженной концентрацией йода составила 50,1%). По данным УЗИ ЩЖ у 17% обследованных детей был выявлен диффузный зоб, частота которого варьировала от 9,4 до 29% по областям исследования. Доля йодированной соли, употребляемой в семьях школьников районов исследования, составила 17,8% (диапазон значений от 15,6 до 19%), что свидетельствует о крайне низком уровне потребления йодированной соли населением. Вся соль, используемая для приготовления пищи в школьных столовых районов исследования, была йодированной, что подтверждает соблюдение требований СанПиН 2.4.5.2409-08.ЗАКЛЮЧЕНИЕ. Несмотря на активное проведение в Брянской области различных профилактических программ йододефицитных заболеваний и социальных мероприятий по пропаганде использования йодированной соли, в условиях отсутствия по настоящее время массовой профилактики при помощи йодированной соли, следует констатировать их неудовлетворительные результаты.

## Abstract (en)

BACKGROUND: The Bryansk region is one of the regions of the Russian Federation most affected by the accident at the Chernobyl nuclear power plant on April 26, 1986.In the conditions of a chronic uncompensated deficiency of iodine in the diet in the first months after the accident, an active seizure of radioactive iodine by the thyroid tissue took place, which inevitably resulted in an increase in thyroid diseases from the population in the future. The article presents the results of a control and epidemiological study carried out in May 2021 by specialists of the National Medical Research Center of Endocrinology of the Ministry of Health of Russia, aimed at assessing the current state of iodine provision in the population of the Bryansk region.AIM: Assessment of iodine supply of the population of the Bryansk region.MATERIALS AND METHODS: The research was carried out in secondary schools of three districts of the Bryansk region (Bryansk, Novozybkov and Klintsy).The study included 337 schoolchildren of pre-pubertal age (8–10 years), all children underwent: measurement of height and weight immediately before the doctor’s examination, which included palpation of the thyroid gland (thyroid gland); Thyroid ultrasound using a portable device LOGIQe (China) with a multi-frequency linear transducer 10–15 MHz; determination of iodine concentration in single portions of urine. A qualitative study for the presence of potassium iodate in samples of table salt (n = 344) obtained from households and school canteens was carried out on the spot using the express method.RESULTS: According to the results of a survey of 337 pre-pubertal children, the median urinary iodine concentration (mCIM) is 98.3 μg / L (range from 91.5 to 111.5 μg / L, the proportion of urine samples with a reduced iodine concentration was 50.1%). According to the ultrasound of the thyroid gland, 17% of the examined children had diffuse goiter, the frequency of which varied from 9.4 to 29% in the areas of study. The share of iodized salt consumed in the families of schoolchildren in the study areas was 17.8% (values range from 15.6 to 19%), which indicates an extremely low level of iodized salt consumption by the population. All salt used for cooking in school canteen areas of the study was iodized, which confirms compliance with the requirements of SanPiN 2.4.5.2409–08.CONCLUSION: Despite the active implementation in the Bryansk region of various preventive programs of IDD and social activities to promote the use of iodized salt, in the absence of mass prevention with the help of iodized salt to date, their unsatisfactory results should be noted.

## ОБОСНОВАНИЕ

Йододефицитные заболевания (ЙДЗ) относятся к числу наиболее распространенных неинфекционных заболеваний во всем мире, представляя собой глобальную угрозу здоровью людей, требующую значительных ресурсов системы здравоохранения на их лечение [1–3]. Наиболее очевидное проявление йододефицита (ЙД) — эндемический (диффузный нетоксический) зоб, являющийся предрасполагающим фактором для развития различных узловых форм заболеваний щитовидной железы (ЩЖ). Спектр йододефицитной патологии обширен и не ограничивается только заболеваниями ЩЖ. Наиболее важными являются нарушение формирования и развития ЦНС плода, прежде всего головного мозга, с ограничением когнитивных и морфофункциональных параметров развития у детей вплоть до выраженных форм умственной отсталости [1][2].

На сегодняшний день очевидно, что ключевая составляющая в достижении успеха в устранении ЙД формируется только при содействии в решении проблемы ЙДЗ органов государственной власти и закреплении принятых решений на законодательном уровне [1–3].

В начале прошлого века ликвидация ЙД была в приоритете задач системы здравоохранения СССР, благодаря чему в 1950–1970-х гг. достигнуты значительные успехи в борьбе с эндемическим зобом. Но после свертывания отлаженной системы противозобных мероприятий был утрачен и системный контроль за распространением ЙДЗ [1–2].

Брянская область относится к числу территорий страны с дефицитом ряда микроэлементов, в том числе йода, в почве и воде. В результате аварии на Чернобыльской атомной электростанции (ЧАЭС) 26 апреля 1986 г. к ЙД в биосфере добавилось и техногенное загрязнение радионуклидами. В течение первого месяца после аварии на ЧАЭС наиболее значимым источником внутреннего облучения был изотоп 131I, который попадал в организм ингаляционно и через потребление в пищу загрязненных продуктов питания. В силу ряда причин своевременных массовых защитных мероприятий по уменьшению поступления радиоизотопов йода в ЩЖ не проводилось среди населения пострадавших регионов РФ. В условиях природного ЙД на территории радиоактивно загрязненных районов отмечалось выраженное поражающее действие радиойода за счет его активного накопления в ЩЖ, что в последующем определило достоверный рост заболеваний ЩЖ у населения, при этом из всех регионов страны Брянская область пострадала в наибольшей степени [4–6].

Так, уже к 1995 г., опираясь на результаты обследования и изучения йодной обеспеченности населения в областях, пострадавших при аварии на ЧАЭС, было установлено наличие зобной эндемии среднетяжелой степени выраженности в Брянской, Калужской, Тульской и Орловской областях, причиной которой стал не только ЙД, но и антропогенные загрязнители. Несмотря на легкий ЙД (медианная концентрация йода в моче (мКЙМ) 69–84 мкг/л), частота диффузного зоба у детей составила 38,6%, при этом отмечалась его ранняя манифестация, начиная с 3-летнего возраста [4][7][8].

Через несколько лет после аварии на ЧАЭС в Белоруссии, а затем в России и Украине стали регистрировать повышенную заболеваемость раком щитовидной железы (РЩЖ) у населения радиационно загрязненных областей, и главной особенностью возрастной группы РЩЖ являлась высокая доля детей. Так, в 1994 г. 7,0% всех больных РЩЖ в Брянской области составляли дети до 14 лет, тогда как в России в целом этот показатель был 1,3% [9]. Обращала на себя внимание и динамика заболеваемости РЩЖ у детей в возрасте до 14 лет: в период 1990–1994 гг. увеличилась с низкого фонового значения до 10 случаев на миллион по Брянской и Калужской областям [10]. Наибольший прирост был выявлен в Брянской области, где с 1986 по 1994 гг. был диагностирован 21 случай РЩЖ у детей, в дальнейшем тенденция не изменилась: с 1986 до 2001 гг. среди детей Брянской области зарегистрировано 49 случаев РЩЖ, в т.ч. по юго-западным районам — 32 случая, что в 24,5 раза выше по сравнению с периодом 1975–1986 гг. (2 случая) [9–11]. Заболеваемость злокачественными новообразованиями детского населения Брянской области в целом выросла с 0 в 1986 г. до 2,6 на 100 тыс. детей в 1994 и 1995 гг., а по юго-западным районам области — с 0 до 10,9 на 100 тыс. детей соответственно [4][5].

Отмечая достоверный рост тиреоидной патологии на территории всей Брянской области и неэффективность предпринимаемых организационных мер по ликвидации негативного влияния ЙД на здоровье жителей, начиная с 1996 г. были разработаны и последовательно внедрены областные целевые программы, направленные на снижение распространенности заболеваний ЩЖ («Предупреждение и профилактика заболеваний щитовидной железы на территории Брянской области» (1996–2000 гг.), «Предупреждение и лечение заболеваний щитовидной железы на территории Брянской области» (2001–2005 гг.), «Минимизация медицинских последствий экологического неблагополучия в Брянской области» (2005–2009 гг.)). В рамках этих программ за счет средств областного бюджета по выборочным районам велась работа по оценке степени ЙД (исследование йодурии) и скринингу на патологию ЩЖ (лабораторное определение тиреоидного статуса, УЗИ ЩЖ), закупались препараты йодида калия для проведения индивидуальной профилактики ЙД среди беременных и кормящих.

Принятое в октябре 1999 г. Постановление Правительства РФ №1119 «О мерах по профилактике заболеваний, связанных с дефицитом йода» установило существующую по настоящее время «добровольную» модель профилактики ЙДЗ, и органам исполнительной власти субъектов РФ было рекомендовано принять меры по насыщению рынка продовольственных товаров продукцией, обогащенной йодом. Во исполнение этого Постановления Правительства РФ с начала 2000-х гг. Администрацией Брянской области был издан ряд региональных нормативно-правовых актов — постановления «О профилактике йодной недостаточности у населения Брянской области» от 22 мая 2000 г. №236, «О профилактике йододефицитных заболеваний на территории области» от 12 августа 2002 г. № 319, «Об обеспеченности населения Брянской области йодированной солью и пищевыми продуктами, обогащенными микронутриентами» от 14 июля 2003 г. №299, «О дополнительных мерах по профилактике йододефицитных заболеваний среди населения Брянской области» от 21 сентября 2004 г. №471). С 2002 г. в области запущено производство йодированных хлебобулочных изделий (в 2002 г. на территории области продано 2604,5 т хлебобулочных изделий с йодказеином, что составило 2,9% от всего объема выпущенного за год хлеба, в 2003 г. — 5294,2 т (4,8%) соответственно). С 2003 г. на двух молочных комбинатах начато йодирование 5–7% молочной продукции. В 2004 г. Администрацией Брянской области и областной Думой принято решение о выделении денежных дотаций для приобретения йодида калия с целью проведения групповой профилактики йодного дефицита среди беременных.

По данным доклада ГУЗ «Брянский клинико-диагностический центр» «Об исполнении программы «Предупреждение и лечение заболеваний щитовидной железы на территории Брянской области» в 2001–2002 гг.» за период 1998–2002 гг. уровень неонатального тиреотропного гормона (ТТГ) у новорожденных соответствовал легкой степени ЙД — ТТГ более 5 мЕ/л 10,6–15,5% (эпидемиологические критерии легкой степени 3–19,9%) (табл. 1). Частота зоба по данным УЗИ составила от 1,3 до 27,3% (Новозыбковский, Стародубский и Клетнянский районы, г. Клинцы), мКЙМ — 52–77 мкг/л [5].

**Table:** Таблица 1. Данные неонатального скрининга на врожденный гипотиреоз в Брянской области: количество выявленных случаев врожденного гипотиреоза за определенный год (с указанием охвата новорожденных скринингом за этот период (в %))

Годы	2000	2001	2002	2003	2004	2005	2006	2007	2008	2009	2010
Обследовано наврожденныйгипотиреоз	10 657	6857	7478	12 379	11 549	11 576	11 765	13 090	14 200	14 180	13 520
% охвата	96,4	62,2	62,7	98,8	92,4	95,9	98	98,5	98,4	98,4	98
Выявлен врожденный гипотиреоз	5	3	2	3	2	3	6	5	3	6	5

Годы	2011	2012	2013	2014	2015	2016	2017	2018	2019	2020
Обследовано наврожденныйгипотиреоз	13 605	14 100	13 470	13 520	13 683	13 570	11 429	11 331	9934	9500
% охвата	98	99	98,2	98	98,8	98	98	99	99	98
Выявлен врожденный гипотиреоз	4	4	4	4	3	6	6	4	2	2

Согласно сведениям госстатотчетности за 2012 г. в структуре заболеваний ЩЖ взрослого населения Брянской области преобладали диффузный нетоксический зоб (ДНЗ) и другая эутиреоидная патология — 53,1%, также регистрируется непрерывная динамика роста заболеваемости ЩЖ. В частности, с 2000 г. общая заболеваемость ДНЗ у взрослых выросла в 2,8 раза и в 2012 г. составляла 26,6 случая на 1000 взрослых, при этом отмечалось неравномерное распределение показателей по области со значительным повышением регистрации заболеваемости в юго-западных районах — в 5,5 раза (92,2 случая на 1000), относительно остальных территорий — рост в 1,8 раза (14 случаев на 1000). Удельный вес первичной заболеваемости ДНЗ взрослого населения по области в 2012 г. составил 10,9% от всех диагностированных в данном году заболеваний ДНЗ, по юго-западным районам области впервые выявленный ДНЗ составил всего 6,5% общей заболеваемости ДНЗ (в 2011 г. — 6,4%, в 2010 г. — 7,7%), на остальных территориях — 16,4%. Такие различия показателей заболеваемости на радиационно «чистых» и загрязненных территориях области спустя более 20 лет после аварии на ЧАЭС объясняются наличием ежегодных скрининговых программ, включающих УЗИ ЩЖ, которые способствуют активному выявлению заболеваний среди взрослого населения [5][12].

У детей Брянской области основное число всех заболеваний ЩЖ приходится на долю ДНЗ (78,6% в 2012 г.). Как и у взрослых, отмечаются территориальные отличия показателей распространенности заболеваний ЩЖ по районам области: заболеваемость ДНЗ по юго-западным территориям в 2012 г. составляла 96,9 случая на 1000 детского населения, по остальным — 19,1 случая. Удельный вес первичной заболеваемости ДНЗ детского населения по области в целом за 2012 г. составил 64,8% всех диагностированных заболеваний ДНЗ, при детализации доли впервые выявленных ДНЗ по юго-западным районам и остальным территориям зафиксированы значения 74,1% и 56,4% соответственно. Синдром врожденной йодной недостаточности в 2012 г. регистрировался у 8–10 из 100 тыс. детей, при этом в 5 раз чаще на юго-западных территориях области; первичная заболеваемость врожденной йодной недостаточностью составляет 25% всех зарегистрированных случаев этого заболевания [12].

Положительные тенденции снижения как общей заболеваемости патологией ЩЖ, так и по отдельным нозологиям структуры заболеваний ЩЖ у взрослых и детей Брянской области наметились с 2015 г. и отражены далее в таблице 2. Однако, по данным на 2017 г., заболеваемость РЩЖ в Брянской области остается наибольшей в РФ — 425,5 на 100 тыс. населения в год [13].

**Table:** Таблица 2. Официальные данные статистической отчетности по Брянской области: общая заболеваемость патологией щитовидной железы (в динамике у взрослых и детей), структура заболеваний щитовидной железы по нозологиям (в динамике у взрослых и детей)

Наименование классов и отдельных болезней	Код по МКБ-10	год
2016	2017	2018	2019	2020
0–14 лет(число зарегистрированных заболеваний на 1000 детского возраста)
Болезни щитовидной железы	Е00–Е07	36,9	34,3	29,7	31,3	27,9
Из них: синдром врожденной йодной недостаточности	Е00	0,1	0,1	0,1	0,2	0,1
эндемический зоб, связанный с йодной недостаточностью	Е01, Е02	3,3	2,7	2,2	1,4	1,0
субклинический гипотиреоз вследствие йодной недостаточности и другие формы гипотиреоза	Е02, Е03	5,8	5,1	3,3	3,8	3,3
другие формы нетоксического зоба	Е04	23,4	22,2	19,5	20,0	19,1
тиреотоксикоз	Е05	0,09	0,07	0,05	0,03	0,05
тиреоидит	Е06	2,2	1,8	1,7	1,8	1,5
15–17 лет(число зарегистрированных заболеваний на 1000 подросткового возраста)
Болезни щитовидной железы	Е00–Е07	96,4	91,1	88,5	79,8	78,0
Из них: синдром врожденной йодной недостаточности	Е00	0,1	0,1	0,1	0,03	0,03
эндемический зоб, связанный с йодной недостаточностью	Е01, Е02	8,5	8,2	6,7	5,3	5,4
субклинический гипотиреоз вследствие йодной недостаточности и другие формы гипотиреоза	Е02, Е03	10,0	8,6	7,9	6,1	6,7
другие формы нетоксического зоба	Е04	55,0	55,3	54,4	51,7	49,9
тиреотоксикоз	Е05	0,4	0,3	0,2	0,1	0,2
тиреоидит	Е06	8,7	8,5	9,2	8,0	8,5
Взрослое население(число зарегистрированных заболеваний на 1000 взрослого населения)
Болезни щитовидной железы	Е00–Е07	58,4	57,4	57,4	57,5	41,0
Из них: синдром врожденной йодной недостаточности	Е00	-	-	-	-	-
эндемический зоб, связанный с йодной недостаточностью	Е01, Е02	7,5	7,1	6,6	6,4	4,6
субклинический гипотиреоз вследствие йодной недостаточности и другие формы гипотиреоза	Е02, Е03	5,3	4,9	4,4	4,8	3,3
другие формы нетоксического зоба	Е04	31,7	32,8	33,5	33,9	24,0
тиреотоксикоз	Е05	1,4	1,5	1,4	1,5	1,0
тиреоидит	Е06	10,3	9,9	9,6	9,7	7,2

В аналитическом отчете, подготовленном в 2021 г. Национальным медицинским исследовательским центром эндокринологии, была дана оценка динамики эпидемиологических показателей тиреоидной патологии у населения РФ за период 2009–2018 гг. Согласно докладу за десятилетний период, отмечается статистически значимый рост распространенности различных форм зоба и тиреотоксикоза у всего населения РФ. В отношении заболеваемости синдромом врожденной йодной недостаточности выявлена лишь тенденция к росту (за 10 лет медиана заболеваемости составила 0,4 случая на 100 000 человек, медиана ежегодного прироста заболеваемости — 0,04 случая на 100 000 человек). Несмотря на то что в течение периода наблюдения число новых случаев различных форм зоба уменьшилось, распространенность зоба среди населения РФ не достигла своего спорадического уровня и остается по-прежнему высокой: 1,2% населения к 1 января 2019 г. [14].

Следует с сожалением констатировать, что организационные мероприятия по борьбе с ЙДЗ в Брянской области имеют неудовлетворительные результаты в виде сохранения относительно высокой распространенности зобной патологии среди взрослого и детского населения, что доказывает недостаточную их эффективность в условиях отсутствия массовой профилактики при помощи йодированной соли.

## ЦЕЛЬ ИССЛЕДОВАНИЯ

Оценка йодной обеспеченности населения Брянской области.

## МАТЕРИАЛЫ И МЕТОДЫ

Исследование проводилось в общеобразовательных школах трех районов Брянской области (гг. Брянск, Новозыбков и Клинцы).

В исследование были включены 337 школьников допубертатного возраста (8–10 лет), всем детям выполнено: измерение роста и веса непосредственно перед осмотром врача, включавшим пальпацию ЩЖ; УЗИ ЩЖ с использованием портативного аппарата LOGIQe (China) с мультичастотным линейным датчиком 10–15 МГц; определение концентрации йода в разовых порциях мочи.

Исследование проводилось одномоментно с 18 по 20 мая.

От всех родителей/опекунов детей получены информированные согласия на проведение обследования и обработку персональных данных. Разрешение локального этического комитета ФГБУ «НМИЦ эндокринологии» Минздрава России получено 25 марта 2020 г., протокол №5.

Место и время проведения исследования

Выполнение работ осуществлялось в период 17–20 мая 2021 г. в следующих районах Брянской области.

1.Город Брянск.

2.Город Клинцы (Клинцовский район).

3.Город Новозыбков (Новозыбковский район).

Изучаемые популяции (одна или несколько)

Одна популяция.

Способ формирования выборки из изучаемой популяции (или нескольких выборок из нескольких изучаемых популяций)

Выбор школ и населенных пунктов выполнялся с учетом количества обучающихся и возможности обследовать не менее 30 школьников в возрасте 8–10 лет.

Выборка была сформирована методом систематического выбора с учетом обучения в школах не только детей, проживающих в данном городе, но и приезжающих из других населенных пунктов региона.

Дизайн исследования

Одномоментное популяционное исследование.

## МЕТОДЫ

Всего в группу исследования были включены 337 детей допубертатного возраста (8–10 лет) (рис. 1) с нормальным физическим развитием. Исследование проводилось с 18 по 20 мая 2021 г. в общеобразовательных школах трех районов Брянской области — в гг. Брянск, Новозыбков и Клинцы (в каждом из районов группы обследуемых были сопоставимы по количеству детей).

**Figure:**
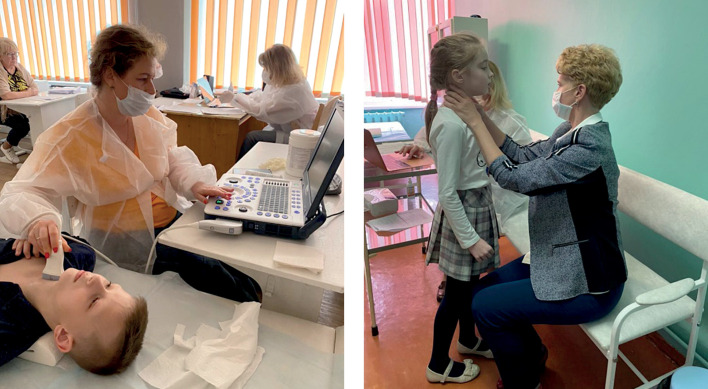
Рисунок 1. Сотрудники ФГБУ «НМИЦ эндокринологии» проводят обследование детей в Брянской области.

Обследование проводилось в соответствии с рекомендациями ВОЗ [17] и включало: сбор анамнеза и оценку антропометрических показателей (рост, вес), осмотр врачом-эндокринологом с пальпацией ЩЖ, УЗИ ЩЖ, определение концентрации йода в разовых порциях мочи. Также проведено определение содержания йода в образцах пищевой поваренной соли, которая ежедневно используется в питании детей в семьях и при приготовлении пищи в столовых общеобразовательных школ.

Измерения роста и веса детей проводились во время осмотра специалистом. УЗИ ЩЖ с определением объема и эхоструктуры выполнялось в положении лежа с использованием портативного ультразвукового аппарата LOGIQe (China) с мультичастотным линейным датчиком 10–15 МГц. Соответствие объема ЩЖ нормативным показателям, разработанным Zimmermann М. и соавт., оценивалось с учетом площади поверхности тела и пола детей [15].

Все образцы мочи (n=337) в одноразовых микропробирках типа Эппендорф сразу же после получения подвергались заморозке при температуре –20–25°! для дальнейшего определения концентрации йода в моче с помощью церий-арсенитного метода (на базе клинико-диагностической лаборатории ФГБУ «НМИЦ эндокринологии» Минздрава России).

Качественное исследование на наличие йодата калия в образцах пищевой поваренной соли (n=344) осуществлялось на месте экспресс-методом, принцип которого заключается в изменении окраски раствора крахмала при выделении свободного йода из соли после обработки ее тест-раствором. Степень изменения окраски оценивается визуально.

От всех родителей/опекунов детей получены информированные согласия на проведение обследования и обработку персональных данных. Разрешение локального этического комитета ФГБУ «НМИЦ эндокринологии» Минздрава России получено 25 марта 2020 г., протокол №5.

Статистический анализ

Данные представлены в виде абсолютных значений и процентов от общего количества. Для описательного статистического анализа концентрации йода в моче были использованы значения медианы и частотного распределения.

Этическая экспертиза

Протокол исследования одобрен на заседании этического комитета ФГБУ «НМИЦ эндокринологии» Минздрава России от 25 марта 2020 г. (протокол № 5).

## РЕЗУЛЬТАТЫ

По результатам обследования 337 детей младшего школьного возраста мКЙМ составила 98,3 мкг/л и варьирует от 91,5 до 111,5 мкг/л в обследованных районах, доля проб мочи со сниженной концентрацией йода составила 50,1% (рис. 2).

Доля йодированной соли, употребляемой в семьях школьников районов исследования, составила 17,8% (диапазон значений от 15,6 до 19%), что свидетельствует о крайне низком уровне потребления йодированной соли населением (рис. 3а, 3б).

**Figure:**
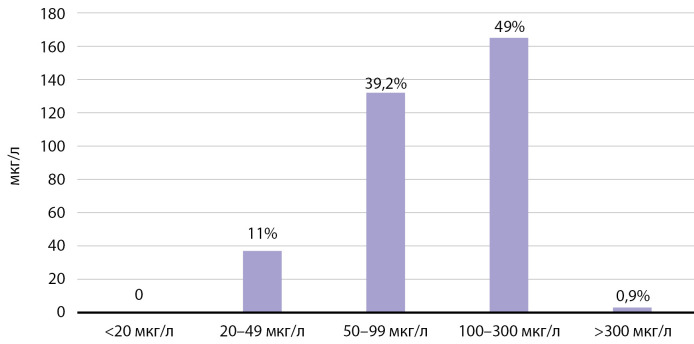
Рисунок. 2. Анализ частотного распределения концентрации йода в моче у обследованных школьников.

**Figure:**
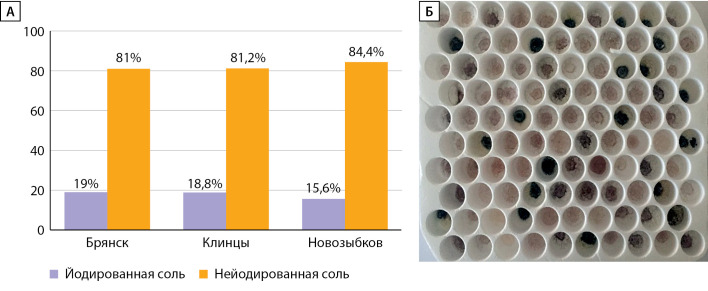
Рисунок 3. А. Результаты определения наличия йода в пищевой соли из домохозяйств. Б. Фото результата экспресс-теста на определение наличия йода в пищевой соли (темно-синее окрашивание образцов соли подтверждает наличие в них йодата калия).

Вся соль, используемая для приготовления пищи в школьных столовых районов исследования, была йодированной, что подтверждает соблюдение требований СанПиН 2.4.5.2409–08 для общеобразовательных учреждений (Постановление Главного государственного санитарного врача РФ от 23.07.2008 г. №45 в редакции от 25.03.2019 г.).

По данным УЗИ ЩЖ у 17% обследованных детей был выявлен диффузный зоб, частота зоба в исследуемой выборке варьировала от 9,4 до 29%.

Пальпаторное определение размеров ЩЖ является доступным методом и при достаточном опыте исследователя обладает хорошим показателем чувствительности в случае высокой распространенности зоба в популяции и/или преобладания больших степеней увеличения ЩЖ. По нашим данным, пальпация ЩЖ показала сопоставимую с УЗИ выявляемость зоба с незначительной переоценкой частоты его распространенности — в среднем при пальпации распространенность диффузного зоба составила 23,3%.

Результаты проведенных исследований в населенных пунктах Брянской области представлены в таблице 3.

**Table:** Таблица 3. Результаты исследований в населенных пунктах Брянской области

Населенный пункт	Распространенностьдиффузного зоба, %	мКЙМ, мкг/л	Доля йодированной соли, %
по данным УЗИ	по данным пальпации
г. Брянск	9,4	10,7	111,5	19
г. Новозыбков	12,5	15,3	92	15,6
г. Клинцы	29	44	91,5	18,8
Среднее значение для Брянской области	16,96	23,3	98,3	17,8

## ОБСУЖДЕНИЕ

Бедность почв Брянской области йодом обуславливает широкое распространение ЙДЗ [8]. В 1986 г. загрязнение техногенными радионуклидами в результате аварии на ЧАЭС значительно увеличило заболеваемость ЩЖ в области, особенно у детей и подростков. С 1996 г. на территории Брянской области были предприняты попытки по устранению ЙД посредством принятия региональных целевых программ, включающих мероприятия по скринингу тиреоидной патологии, обогащению продуктов питания йодом и индивидуальной профилактике среди декретированных контингентов, пропаганде использования йодированной соли. Однако результаты проведенного анализа свидетельствуют о том, что даже комплекс перечисленных мер не обладает достаточной эффективностью и проблема ЙДЗ остается нерешенной — в настоящее время в Брянской области нет действующей программы массовой профилактики ЙДЗ.

Результаты очередного мониторинга показывают сохранение дефицита йода в питании населения Брянской области и в целом совпадают с выявленными тенденциями и выводами сопоставимых исследований, выполненных по аналогичному дизайну в Республике Крым и Республике Тыва в 2020 г. [16][17].

В Брянской области, на фоне крайне низкого уровня использования пищевой йодированной соли в домохозяйствах (17,8%), умеренное снижение мКЙМ (98,3 мкг/л) не позволяет считать потребление йода достаточным для всего населения региона. Соблюдение требований СанПиН 2.4.5.2409–08, устанавливающего обязательное использование йодированной соли в школьном питании с начала 2020 г., подтверждено проверками соли в школьных столовых районах исследования, в связи с чем потребление йода и показатели его экскреции с мочой у детей школьного возраста могут быть несколько выше истинного уровня и неточно отражать обеспеченность йодом всей популяции.

Степень тяжести ЙДЗ в Брянской области, определяемая на основании показателей величины экскреции йода с мочой и распространенности зоба у детей по данным УЗИ, соответствует эпидемиологическим критериям легкой степени тяжести (до 99 мкг/л и 19,9% по соответствующим критериям). При этом следует отметить, что степень выраженности йодного дефицита у школьников, проживающих в менее крупных населенных пунктах, была более значительной по сравнению с городскими жителями. Так, наиболее неблагоприятная эпидемиологическая ситуация выявлена у детей Клинцовского района: мКЙМ — 91,5 мкг/л, распространенность зоба — 29%, тогда как у городского населения Брянска эти показатели лучше: 111,5 мкг/л и 9,4% соответственно. Наиболее вероятным объяснением большей частоты зоба у детей Клинцовского района является предположение разницы пищевых рационов жителей областей. Если в питании городского населения преобладают продукты промышленного производства, обогащенные микроэлементами, и «привозные» морепродукты, то жители сельской местности в большей степени традиционно употребляют в пищу продукты местного происхождения (в том числе самостоятельно выращенные/полученные).

Также обращает на себя внимание величина распространенности зобных изменений ЩЖ в Брянской области (до 29%) на фоне диапазона значений мКЙМ от 91,5 до 111,5 мкг/л. В формировании зобной эндемии, очевидно, помимо ЙД, могут участвовать и другие факторы, что подводит к необходимости проведения дальнейших эпидемиологических исследований с включением большего числа участников и районов, а также углубленного сравнительного анализа с данными по другим регионам РФ [17].

Пальпаторная оценка размеров ЩЖ у детей, приводящая к гипердиагностике размеров зоба в условиях легкой степени йодной недостаточности, очевидно, не может быть достаточным методом для выявления ЙДЗ в рамках критериев проведения эпидемиологических обследований. При этом нельзя упускать доступность и продемонстрированную чувствительность методики, которая является ценным инструментом для врачей, определяя показания для дополнительных методов обследования.

Разработка региональной профилактической программы, ее реализация и регулярный мониторинг эффективности, пропаганда использования йодированной соли и повышение общественной информированности о проблемах, ассоциированных с дефицитом йода, безусловно, являются важными составляющими комплекса медико-социальных мероприятий по ликвидации заболеваний, связанных с дефицитом йода в Брянской области.

Клиническая значимость результатов

Клиническая значимость полученных результатов состоит в комплексной оценке состояния йодной обеспеченности и зобной эндемии и необходимости принятия программ профилактики ЙДЗ на территории Брянской области.

Направления дальнейших исследований

В продолжение работы, при согласовании с Департаментом здравоохранения Брянской области, планируются проведение исследований с включением других районов области и сопоставление полученных данных с результатами неонатального скрининга на врожденный гипотиреоз, а также исследования йодной обеспеченности беременных и кормящих женщин на фоне массовой йодной профилактики.

## ВЫВОДЫ

Результаты эпидемиологического исследования свидетельствуют о недостаточной обеспеченности йодом населения Брянской области: показатель мКЙМ составляет 98,3 мкг/л, поскольку доля домохозяйств, использующих йодированную соль, крайне низкая (17,8%), что не соответствует рекомендациям ВОЗ для регионов с природным дефицитом йода (от 90% и более).

В целом для Брянской области характерна легкая степень тяжести ЙДЗ с частотой распространения зоба у детей по данным УЗИ в среднем 17% (варьировала от 9,4 до 29%).

Анализ полученных в ходе обследования данных выявил характерные для йододефицитных регионов закономерности: с нарастанием степени йодной недостаточности увеличивается частота зоба у детей младшего школьного возраста (наиболее неблагоприятная эпидемиологическая ситуация зафиксирована в Клинцовском районе, где доля зоба у детей составила 29%, при мКЙМ 91,5 мкг/л и низкой доле йодированной соли — 18,8%).

Проявления зобной эндемии более выражены у детей, проживающих в менее крупных населенных пунктах, чем у городских жителей (зоб по УЗИ выявлен у 29% и 9,4% детей соответственно), в этих же населенных пунктах уровень использования йодированной соли в пище был ниже.

## ЗАКЛЮЧЕНИЕ

Контрольно-эпидемиологическое исследование, проведенное ФГБУ «НМИЦ эндокринологии» Минздрава России в 2021 г. с целью оценки текущей ситуации по йодной обеспеченности населения Брянской области, подтвердило, что, несмотря на принимаемые меры, сохраняется дефицит йода в питании. Таким образом, ЙД продолжает оказывать негативное влияние на здоровье детей, неизбежно приводя к крайне неблагоприятным последствиям для всего населения региона, пострадавшего от радиоактивного загрязнения после аварии на Чернобыльской АЭС.

Выявленное в Брянской области неадекватное потребление йода в очередной раз демонстрирует неэффективность существующей в России «добровольной модели» профилактики ЙДЗ: наличие ЙД легкой степени и зобной эндемии у детей на фоне крайне низкого использования йодированной соли в домохозяйствах.

Отечественный и мировой опыт борьбы с ЙДЗ свидетельствует, что единственно действенной мерой по ликвидации дефицита йода в питании является массовая профилактика йодированной солью.
